# Toolboxes for a standardised and systematic study of glycans

**DOI:** 10.1186/1471-2105-15-S1-S9

**Published:** 2014-01-10

**Authors:** Matthew P Campbell, René Ranzinger, Thomas Lütteke, Julien Mariethoz, Catherine A Hayes, Jingyu Zhang, Yukie Akune, Kiyoko F Aoki-Kinoshita, David Damerell, Giorgio Carta, Will S York, Stuart M Haslam, Hisashi Narimatsu, Pauline M Rudd, Niclas G Karlsson, Nicolle H Packer, Frédérique Lisacek

**Affiliations:** 1 Biomolecular Frontiers Research Centre, Macquarie University, Sydney, 2109, Australia; 2 Complex Carbohydrate Research Center, University of Georgia, Athens, 30602, USA; 3 Institute of Veterinary Physiology and Biochemistry, Justus-Liebig-University Giessen, Giessen, 35390, Germany; 4 Proteome Informatics Group, SIB Swiss Institute of Bioinformatics, Geneva, 1211, Switzerland; 5 Department of Biomedicine, Gothenburg University, Gothenburg, 405 30, Sweden; 6 Department of Bioinformatics, Soka University, Tokyo, 192-003, Japan; 7 Molecular Biosciences, Imperial College, London, SW7 2AZ, UK; 8 GlycoScience Group, NIBRT, Dublin, Ireland; 9 Research Center for Medical Glycoscience, National Institute of Advanced Industrial Science and Technology, Tsukuba, 305-8568, Japan; 10 Centre Universitaire de Bioinformatique, University of Geneva, Geneva, 1211, Switzerland; 11 Structural Genomics Consortium, University of Oxford, Oxford, OX3 7DQ, UK

## Abstract

**Background:**

Recent progress in method development for characterising the branched structures of complex carbohydrates has now enabled higher throughput technology. Automation of structure analysis then calls for software development since adding meaning to large data collections in reasonable time requires corresponding bioinformatics methods and tools. Current glycobioinformatics resources do cover information on the structure and function of glycans, their interaction with proteins or their enzymatic synthesis. However, this information is partial, scattered and often difficult to find to for non-glycobiologists.

**Methods:**

Following our diagnosis of the causes of the slow development of glycobioinformatics, we review the "objective" difficulties encountered in defining adequate formats for representing complex entities and developing efficient analysis software.

**Results:**

Various solutions already implemented and strategies defined to bridge glycobiology with different fields and integrate the heterogeneous glyco-related information are presented.

**Conclusions:**

Despite the initial stage of our integrative efforts, this paper highlights the rapid expansion of glycomics, the validity of existing resources and the bright future of glycobioinformatics.

## Background

Glycans or carbohydrates, both in the form of polysaccharides or glycoconjugates are known to partake in many biological processes and increasingly recognised as being implicated in human health. Glycosylation is probably the most important post-translational modification in terms of the number of proteins modified and the diversity generated. Since glycoproteins, glycolipids and glycan-binding proteins are frequently located on the cell's primary interface with the external environment many biologically significant events can be attributed to glycan recognition. In fact, glycans mediate many important cellular processes, such as cell adhesion, trafficking and signalling, through interactions with proteins. Protein-carbohydrate interactions are also involved in many disease processes including bacterial and viral infection, cancer metastasis, autoimmunity and inflammation [1-3].

In spite of such a central role in biological processes, the study of glycans remains isolated, protein-carbohydrate interactions are rarely reported in bioinformatics databases and glycomics is lagging behind other -omics. However, a key impetus in glycomics is now perceptible in the move toward large-scale analysis of the structure and function of glycans. A diverse range of technologies and strategies are being applied to address the technically difficult problems of glycan structural analysis and subsequently the investigation of their functional roles, ultimately to crack the glycocode.

Adding meaning to large data collections requires advances in software and database solutions, along with common platforms to allow data sharing. Current glycobioinformatics resources do cover information on the structure and function of glycans, their interaction with proteins or their enzymatic synthesis. However, this information is partial, scattered and often difficult to find for non-glycobiologists.

Several initiatives to catalogue and organise glycan-related information were launched in the past couple of decades starting with CarbBank [4,5] in 1987. Regrettably, funding for this structural database was discontinued in 1997. Several projects have followed, among which EUROCarbDB [6] is the most recent, though now also unfunded since 2011. In many cases, these databases have remained confined to the realm of glycoscientists and their restricted popularity has often led to the withdrawal of funds. A similar fate is awaiting the databases created by the Consortium for Functional Glycomics (CFG) [7] despite twelve years of service but with limited connectivity to other leading bioinformatics resources such as those hosted at NCBI http://www.ncbi.nlm.nih.gov, EBI http://www.ebi.ac.uk or on the ExPASy server http://www.expasy.org to name only a few.

Even though a few stable initiatives such as GlycomeDB [8,9], or KEGG-GLYCAN [10] have remained, the bleak prospect of producing yet another resource as part of yet another rescue plan likely to collapse a few years later, led our small but dedicated glycobioinformatics community to adopt cooperative strategies for enhancing the consistency of existing online services, and bridging with other -omics initiatives, thereby bringing glycomics to the fore. The development of compatible and complementary toolboxes for analysing glycomics data and cross-linking results with other -omics datasets appears as a solution to longer-term prospects and stability.

An obstacle in linking glycomics with other -omics is the independent accumulation of data regarding the constituents of glycoconjugates. Few protocols have been developed that produce data for the glycan, the glycoconjugates and their relationship to each other to allow the generation of datasets containing information from both perspectives. In fact, most glycan structures have been solved after being cleaved off their natural support (e.g., glycoproteins or glycolipids). Consequently, key information on the conjugate is lost. Conversely, protein glycosylation sites are studied and stored independently of the sugar structure [11] that is often not solved in the process. As a result, key information on the attached glycan structures is lost. The correlation between glycan structures and proteins can sometimes be partially restored manually through literature searches that are both labour and time consuming. Nonetheless, the expansion of systems biology that brings together multiple aspects of a biological phenomenon is steadily integrating glycomics data. Recently, this approach was followed in a study by Lauc and colleagues, to unveil the role of glycans in immunity [12].

This paper (1) reviews the extent of previously defined standards for representing glycan-related information and its consequences for automated analysis, (2) describes existing software for solving some of the issues raised in (1), (3) emphasises the means of cross-linking glycobioinformatics and other bioinformatics resources and (4) highlights collaborative efforts of integration within glycomics applications. Our report highlights the benefit of including glycomics to better understand biological processes and the necessary steps to achieve this goal.

## Methods

### Specific issues of glycan representation

#### *Nomenclatures and formats*

Nucleic acids and proteins can be represented (at least in their most basic forms) as simple character strings. In contrast, glycans are inherently more complex and involve significant degrees of branching. Moreover, the breadth of monosaccharide diversity (the building blocks of glycans) compared to the 4 nucleotides of nucleic acids and the 20 amino acids of proteins is substantially more extensive. Even though the mammalian glycome seems to arise from approximately 20 monosaccharides [13], bacterial glycans show more than ten-fold greater diversity at the monosaccharide level, and a nine-fold difference at the disaccharide unit space [14]. Consequently, a simple textual representation of this complexity that should include monosaccharide anomericity, glycosidic linkages, residues modifications and substitutions, and account for structure ambiguity is difficult to capture in a format akin to those in proteomics and genomics.

To provide a systematic naming for monosaccharides the International Union of Pure and Applied Chemistry (IUPAC) recommended a naming scheme [15], which was last updated in 1996. However, glycobiology is no better than any other field in the life sciences with an increasing collection of nomenclatures and terminologies expressing redundant chemical formulas and/or molecule or residue names. For example, N-acetylneuraminic acid can be symbolised by Neu5Ac or NANA or 2-keto-5-acetamido-3,5-dideoxy-d-glycero-d-galactononulosonic acid and is sometimes referred to as sialic acid. The latter term, however, actually is generic for all variations of neuraminic acid, such as Neu5Ac, Neu5Gc, Neu7Ac, etc. Although Neu5Ac is the most frequently found sialic acid these terms should not be used as synonyms, as they are not equivalent. In addition to these names used in the literature, different names are used in databases, as illustrated in Table 1 that spans existing terms referring to monosaccharide α-**D**-Neu*p*5Ac. As mentioned below in relation to formats, some representations of glycan residues are split into two parts, i.e., monosaccharide and substituent (chemical modification of the monosaccharide) that are described separately.

**Table 1:** Encoding of IUPAC monosaccharide α-D-Neup5Ac in databases

Database	Monosaccharide	Substituent
CarbBank	D-gro-a-D-3-deoxy-galNon2ulop5NAc-onic	
CarbBank	a-D-Neup5Ac	
GLYCOSCIENCES.de	D-gro-a-D-3-deoxy-galNon2ulop5NAc-onic	
GLYCOSCIENCES.de	a-D-Neup5Ac	
GlycomeDB	a-dgro-dgal-NON-2:6|1:a|2:keto|3:d	(5d-1) n-acetyl
UniCarbKB	a-dgro-dgal-NON-2:6|1:a|2:keto|3:d	(5d-1) n-acetyl
CFG	NNa	
BCSDB	aXNeup	(5-1) Ac
Protein Data Bank	SIA	
Protein Data Bank	NAN	

Overview of how the IUPAC monosaccharide α-**D**-Neu*p*5Ac is encoded in different databases as provided by MonosaccharideDB http://www.monosaccharidedb.org. The names of the database (Database), the monosaccharide name (Monosaccharide) and when specified, the separately handled substituent (Substituent), are given in the respectively designated columns. Note that in some databases even different names can be used for the same monosaccharide.

**Figure 1:**
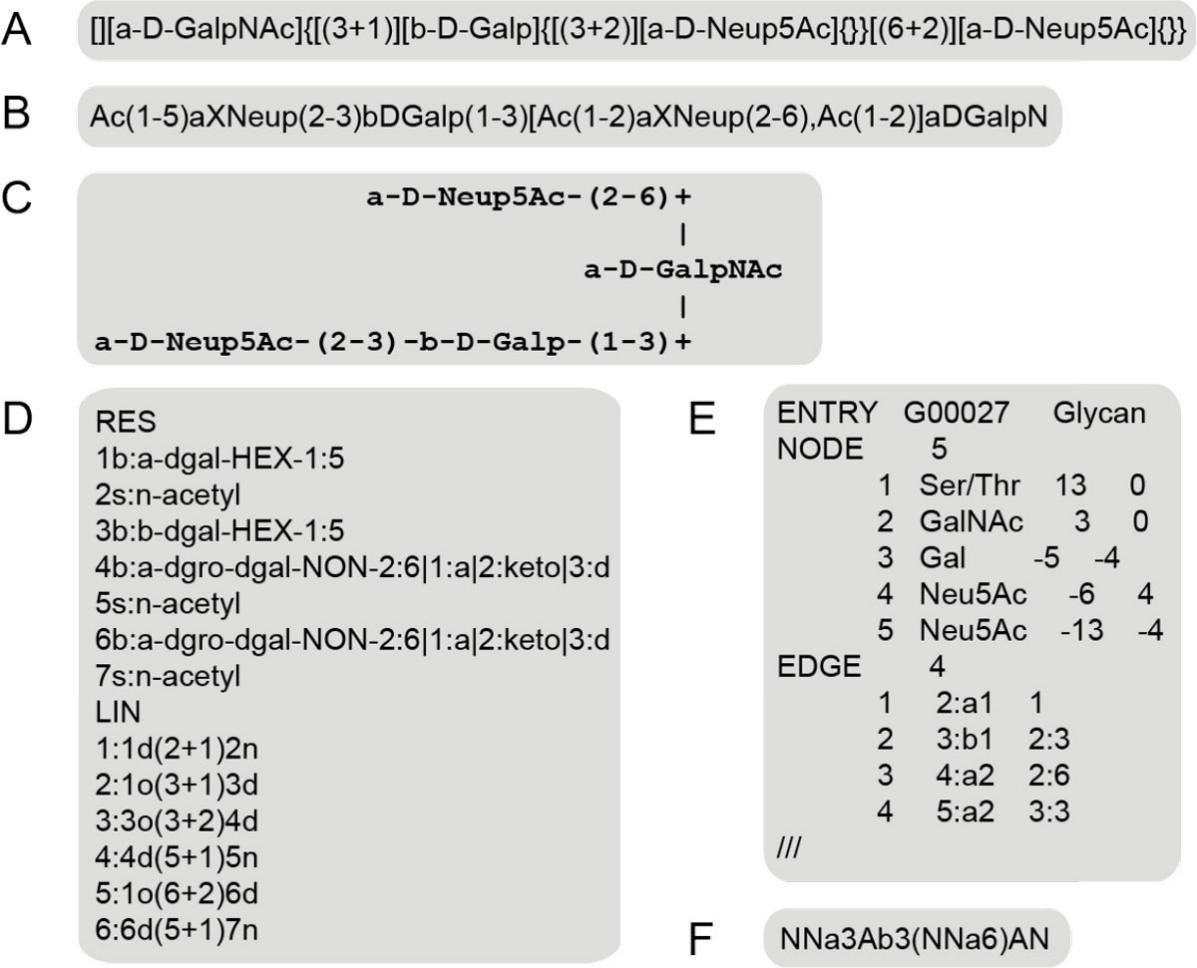
**Encoding formats for glycans**. Examples for the sequence encoding of the O-Glycan with the GlycomeDB ID 534/EUROCarbDB/UniCarbDB ID 598. (A) LINUCS sequence format as used in GLYCOSCIENCES.de. (B) BCSDB sequence encoding. (C) CarbBank sequence format. (D) GlycoCT sequence format as used in GlycomeDB and UniCarbDB. (E) KCF format used in the KEGG database. (F) LinearCode^® ^as used in the CFG database.

Besides the diverse encoding of monosaccharides the next issue is the non-linear nature of glycans, which in the past led to the development of different formats capable of handling this level of complexity in computer applications. In most cases tree-like structures have been linearised using different approaches to encode the inherent complexity of branching. Several examples are depicted in Figure 1 comprising/including the LINUCS format (Figure 1A) [16], the Bacterial Carbohydrate Structure Database sequence format (Figure 1B) [17] and the LinearCode^® ^[18] adopted by the CFG database (Figure 1F). Most of these formats provide rules for sorting of the branches to create a unique sequence for a glycan. Older databases, such as CarbBank, applied a multi text line representation of the structure that resembles the corresponding IUPAC recommendation (Figure 1C). More recent databases have decided upon connection table approaches to bypass the limitations of the linear encodings exemplified by GlycoCT (Figure 1D) [19] and KCF (KEGG Chemical Function) formats (Figure 1E) [20]. Alternative XML based formats, such as CabosML [21] and GLYDE [22], have been defined to be used for exchange of glycan structure information (not shown in Figure 1).

#### *Graphical representations*

**Figure 2:**
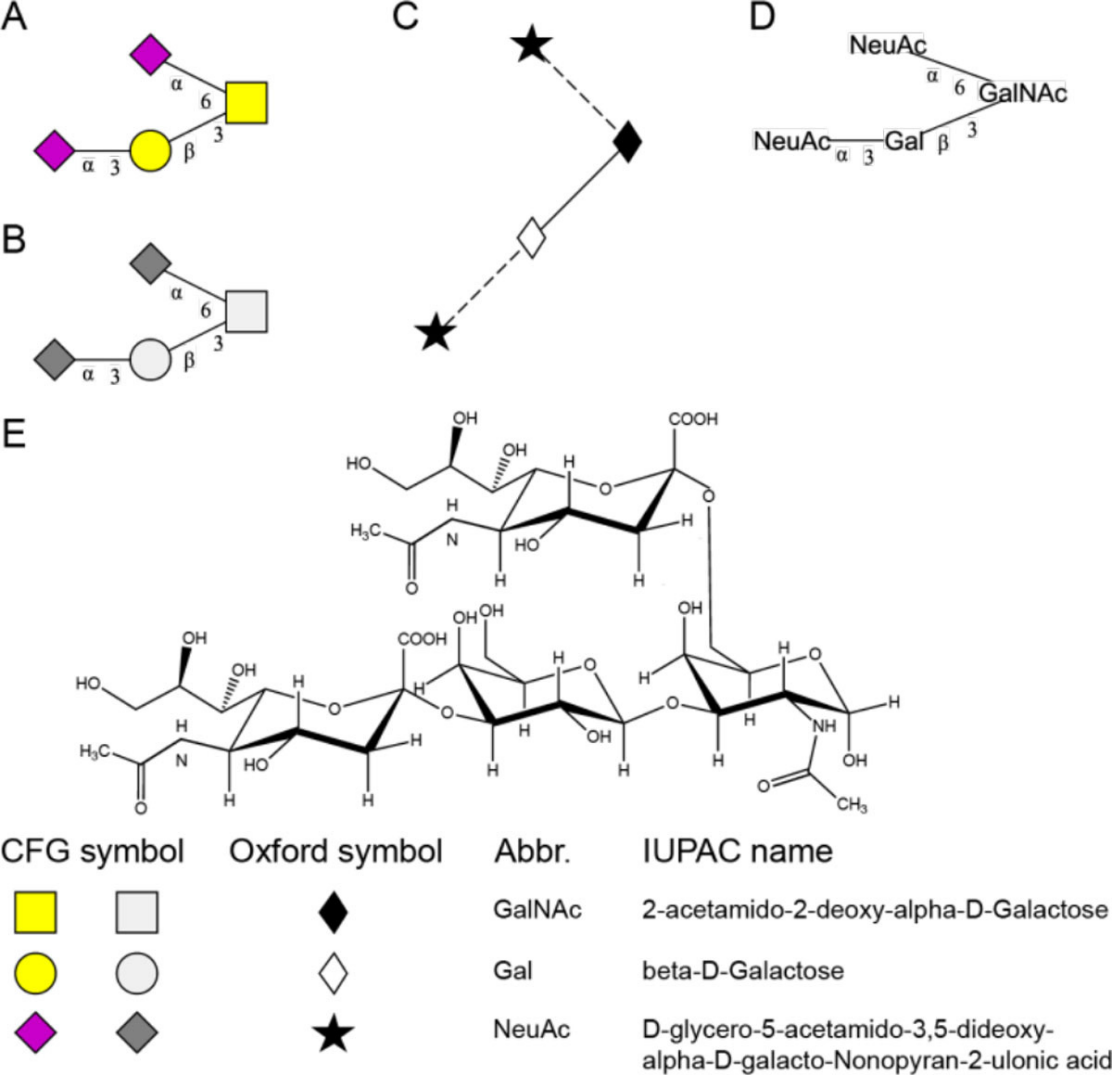
**Graphical representation of glycans**. Examples for graphical representations of glycan structures corresponding to the illustration of Figure 1. (A) CFG cartoon representation using colour symbols. (B) CFG cartoon representation using greyscale symbols. (C) Oxford cartoon representation. (D) IUPAC like representation using textual names. (E) Chemical representation preferred by carbohydrate chemists. A definition of the symbols and corresponding monosaccharide names is shown below (E).

Admittedly, formats shown in Figure 1 are manifestly machine-readable but do not really meet user-friendliness standards. Consequently, in addition to the sequence encoding of structures, several graphical representations are supported by a majority of glyco-focused databases. Figure 2 shows several graphical representations of the glycan described in Figure 1. Here, the monosaccharide names are replaced by symbolic representations, so called cartoons (Figure 2A-C), or by depictions of the chemical structure (Figure 2E). Examples of cartoons are the representation scheme developed by the "Essentials in Glycobiology" textbook editors (and adopted by the CFG) (Figure 2A, B) [2] and the scheme developed by the Oxford Glycobiology Institute [23,24] (Figure 2C). A combination of both these formats in which the linkages are depicted as angles as in the Oxford scheme on the residue symbols of the 'Essentials in Glycobiology' scheme can also be found in web interfaces (such as UniCarbKB) and publications (not shown in Figure 2). In many scientific articles graphics following the monosaccharide names and linkages as defined by the IUPAC nomenclature (Figure 2D) are used. The chemical representation of the glycan (Figure 2E) is preferred by groups that work on the synthesis of glycan structures or on glycan analysis by NMR. EUROCarbDB, GlycomeDB and UniCarbKB [25] have implemented user-interface features that enable switching between supported graphical formats. This feature is made possible by integrating GlycanBuilder [26,27], a tool developed in partnership with EUROCarbDB, which produces graphical representations of glycan structures (see below for more details).

### Adopting standards for representing glycan-related information

One of the common reasons for the development of new sequence encoding formats, instead of adopting existing efforts, is that few initiatives have provided published and documented application programming interfaces (APIs) for parsing and encoding glycan structures. Furthermore, not all formats cover the complete namespace of residues, and each has limitations in the encoding of specific structural features or annotations, such as repeating units or partially missing linkage information as a result of incomplete structure elucidation from acquired experimental data, or limitation of the experimental technique(s) used.

The list of resources cited throughout this report is documented in Table 2 including URLs and a brief content description.

**Table 2:** Current on-line resources

Resource name	Rough content	URL
KEGG-GLYCAN	Glycan structures, References to reactions and pathways, Glyco-gene information	http://www.genome.jp/kegg/glycan
CFG	Glycan structures, MS profile, GlycanArray data, Glyco-gene expression data	http://www.functionalglycomics.org
GlycomeDB	Glycan structures, Cross references	http://www.glycome-db.org
GLYCOSCIENCES.de	Glycan structures, 3D structures, NMR data, Software tools	http://www.glycosciences.de
MonosaccharideDB	Repertoire of monosaccharides	http://www.monosaccharidedb.org
UniCarb-DB	Glycan structures, LC/MS-MS, HPLC data	http://unicarb-db.biomedicine.gu.se
GlycoSuiteDB	Literature based curated glycan structures	http://glycosuitedb.expasy.org
UniCarbKB	Curated glycan structures	http://unicarbkb.org
GlycoBase	Glycan structures, HPLC profiles	http://glycobase.nibrt.ie
JCGGDB	Glycan structures, glyco-gene information, glycomics-related protocols, cross-references to other national life science resources	http://jcggdb.jp/index_en.html
BCSDB	Bacterial glycan structures, NMR data,	http://csdb.glycoscience.ru/bacterial/
RINGS	Software tools	http://rings.t.soka.ac.jp
GlycO ontology	Curated glycan structures	http://bioportal.bioontology.org/ontologies/3169

List of resources cited throughout the text, with corresponding rough content description and URLs.

#### *Accounting for existing software: parsers and translators*

In the past, problems of inconsistent naming of monosaccharides and heterogeneous sequence formats in databases made it almost impossible for users and for bioinformatics software to access and reconcile data from several databases. Therefore several projects including GlycomeDB and RINGS [28] have started developing translation tools for parsing and translating sequence formats from different databases. These translators make it possible to use the databases for statistical analysis [13] and for the mash-up and comparison of data from different sources [14]. It has also led to the creation of the GlycomeDB, a data warehouse for glycan structures that accesses the structural content of almost all publically available glycan structure databases and translates the sequences into a consistent representation creating an index of available glycan structures.

Several tools for the translation of glycan sequence formats have been created by different initiatives including GLYCOSCIENCES.de [29], GlycomeDB, UniCarbKB and RINGS. These are listed in Table 3 in reference to the resources listed in Table 2. Note that import and export functions of GlycanBuilder can also be used for sequence format translation.

**Table 3:** Available format translation across databases

Output → Input↓	IUPAC	KCF	LINUCS	Linear Code	GlydeII	GlycoCT
**IUPAC**		RINGS	GLYCOSCIENCES.de, GlycomeDB, GlycanBuilder	Glycan Builder	GlycomeDB, Glycan Builder	GlycomeDB, UniCarbKB*, Glycan Builder
**KCF**	UniCarbKB*		RINGS, GlycomeDB	RINGS	GlycomeDB, RINGS	GlycomeDB, RINGS
**LINUCS**	GLYCOSCIENCES.de	RINGS		Glycan Builder	GlycomeDB, Glycan Builder	GlycomeDB, Glycan Builder
**Linear Code**		RINGS	GlycomeDB, GlycanBuilder		GlycomeDB, Glycan Builder	GlycomeDB, Glycan Builder
**Glyde II**	-	RINGS	GlycomeDB	-		GlycomeDB
**GlycoCT**	UniCarbKB*	RINGS	GlycomeDB, GlycanBuilder	Glycan Builder	GlycomeDB, Glycan Builder	

Summary of existing software for parsing and translating the most popular formats (partly illustrated in Figures 1 and 2) and the web resource (described in Table 2) where the tool can be run or downloaded. For instance, a LINUCS (Figure 2A) structure can be translated to KCF (Figure 2E) in the RINGS website. Input formats are listed in the rows, and the output formats in the columns. The asterisk indicates that the parsers/translators are available on request but not directly accessible yet as on other websites.

As illustrated above, one of the basic problems for the sequence parsing and translation is the usage of different naming schemes for monosaccharides. For that purpose MonosaccharideDB http://www.monosaccharidedb.org was developed as a web portal and as a programming/lookup library that is used by several of the translation tools for the normalisation and translation of monosaccharide names.

#### *Accounting for existing software: graphical structure input tools*

To access the structural content captured in many databases, web interfaces can be used to search and retrieve information. Early databases such as CarbBank and GLYCOSCIENCES.de were using textual input tools for the structure input making it sometimes difficult for inexperienced users to enter a valid query.

In recent years, graphical input tools have been developed to allow the definition of structures by using the cartoon representation as previously described and illustrated in Figure 2. A majority of existing databases provide users with the tools to search for a defined structure and/or structures containing a substructure. A few databases also allow searching for structure based on well-known motifs or by structural similarity.

**Figure 3:**
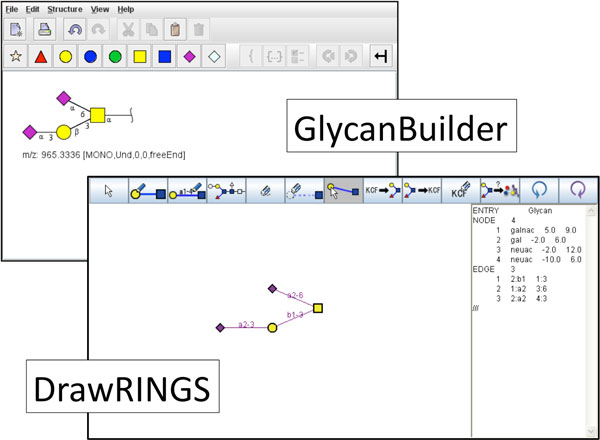
**Graphical interfaces for glycan drawing**. Examples for the output of tools for graphical input corresponding to the illustration of Figure 2. In the fore, a screenshot of DrawRINGS and in the back, a screenshot of GlycanBuilder are shown.

The two most widely used tools for graphical input of glycan structures are GlycanBuilder and DrawRINGS [28]. GlycanBuilder is a web based application that allows the input of glycan structures in all cartoon notations shown in Figure 3 and is also integrated into the GlycoWorkBench software suite that is used for the interpretation of mass spectrometry data [27,30]. DrawRINGS is a JavaApplet that searches for already-known glycan structures that are similar to the drawn glycan structure. In addition, DrawRINGS supports the conversion of KCF encoded structures and supported graphical formats.

#### *Associated semantics*

GlycO [31,32] is a curated ontology that has been developed for representing glycan and glycoconjugates together with their components and their relationship. This ontology is used in combination with other ontologies to model the reactions and enzymes involved in the biosynthesis and modification of glycan structures, and the metabolic pathways in which they participate. Since the glycan structures in the ontology have been added by a multistep manual expert curation, the ontology is also used for the annotation of experimental data.

The GlycO schema relies on the web ontology language and description logic (OWL-DL) to place restrictions on relationships, thus making it suitable to classify new instance data. These logical restrictions are necessary due to the chemical nature of glycans, which have complex, branched structures that cannot be represented in any simple way. The structural knowledge in GlycO is modularized, in that larger structures are semantically composed of smaller canonical building blocks. In particular, glycan instances are modelled by linking together several instances of canonical monosaccharide residues, which embody knowledge of their chemical structure (e.g., β-**D**-Glc*p*NAc) and context (e.g., attached directly to an Asn residue of a protein). This bottom-up semantic modelling of large molecular structures using smaller building blocks allows structures in GlycO to be placed in a biochemical context by describing the specific interactions of its component parts with proteins, enzymes and other biochemical entities.

#### *Experimental data*

An important aspect of glycomics analysis is that very often a single type of experiment is not sufficient to fully define a glycan structure. Orthogonal strategies are employed to fully elucidate structures with a greater measure of confidence. Data acquired from different analytical methods such as NMR, HPLC, MS, glycan array, capillary electrophoresis, monosaccharide analysis or molecular dynamics simulations can be used in combination to characterise complex biological samples. Each experiment solves parts of the puzzle and by combining the derived information from the different experiments it is possible to improve the annotation accuracy. In those cases where the complete structure is not elucidated, due to limits in the experimental methods and acquired data, it is possible to infer some structural features from knowledge about biosynthetic pathways. This is a major difference to classical molecular biology fields, in that the proteome has a template and the genome can now be easily sequenced, whereas the glycome is indirectly encoded via the expression profile of glycosyltransferases, other enzymes involved in glycan synthesis and nucleotide sugar substrate concentrations. However, as in the other -omics initiatives, a concerted effort to define a standard spanning the Minimum Information Required for A Glycomics Experiment (MIRAGE) was initiated in 2011 by some of the authors [33]. Currently only a few databases allow the storage and retrieval of experimental data. In addition most of these databases store only experiments generated by the research group or consortium providing the database. Example databases storing experimental data are EUROCarbDB (MS, NMR, HPLC), GlycoBase in Dublin, Ireland [34] (HPLC), GlycoBase in Lille, France [35] (NMR) and the CFG database (MS profile, Glycan Array). As mass spectrometry (MS) has now become the most common method for solving glycan structures and identifying glycopeptides, there is now an increasing range of software tools that are available for analysing MS data produced in glycomics [36]. At this stage, there is still a low level of integration with other data that needs a joint effort to support workflow creation and integration of MS data analysis. The involvement of some of the authors in the development of UniCarb-DB [37], the first LC MS/MS data repository for glycans is a step in this direction.

## Results

### Bridging with other fields

Adopting standards is a necessary but not a sufficient step towards automating the analysis of glycans. A critical feature/component in glycobioinformatics is the availability of standardised approaches to connect remote databases. The NAS (National Academy of Sciences) "Transforming Glycoscience: A Roadmap for the Future" report [3] exemplifies the hurdles and problems faced by the research community due to the disconnected and incomplete nature of existing databases. Several initiatives have commenced to bridge the information content available in the described databases.

#### *Bridging chemistry and biology with data curation*

GlycoSuiteDB [38,39] contains glycan structures derived from glycoproteins of different biological sources that have been described in the literature, and free oligosaccharides isolated from biologically important fluids (e.g., milk, saliva, urine). The curated database provides contextual information for glycan structures attached to proteins and re-establishes the frequently lost connection between a glycan structure and the attached functional protein as annotated in the UniProtKB resource that is cross-referenced to GlycoSuiteDB. This database is forming the basis of the central glycan structural database in UniCarbKB, which is designed to incorporate information from other structural databases including EUROCarbDB, UniCarb-DB and GlycoBase. The content and manual curation principles of GlycoSuiteDB will form the basis of the central glycan structural database of UniCarbKB to maintain the quality of information stored in the knowledgebase. The links to UniProtKB will help to connect key information between glycosylated sites and specific structures.

#### *Bridging glycobioinformatics and bioinformatics using web services*

The development of a web services protocol enables searches across several databases. Such technologies have gained much attention in the field of life sciences as an open architecture that facilitates interoperability across heterogeneous platforms. An ongoing programme in the glycomics domain is the Working Group on Glycomics Database Standards (WGGDS) activity, which was initially supported by a CFG-bridging grant. A working draft of the protocols can be accessed at http://glycomics.ccrc.uga.edu/GlycomicsWiki/Informatics:Cross-Database_Search/Protocol_%28WGGDS%29. The WGGDS enabled developers from the CFG, EUROCarbDB/UniCarb-DB, GlycomeDB, GLYCOSCIENCES.de and RINGS to seed the beginnings of a communication interface, which provides access to the data contained in multiple, autonomous glycomics databases with an emphasis on structural data collections.

A complete suite of representational state transfer (REST) based tools has been developed by some of the authors with new and improved applications being built. Each service provides access to a (sub-)structure search that supports remote queries for complete or partial structure and allows for substructure/epitope matching. This can only be achieved with universal acceptance of structure encoding formats and access to accurate and complete glycan translators. Here, the sequence attribute of the XML-based message protocol conforms to the GlydeII format (see above), which can be readily converted into GlycoCT and/or KCF formats for executing database searches. In addition, individual databases have expanded this service to enable searching based on molecular mass, experimental evidences, e.g. mass spectrometry, and monosaccharide composition. To realise this goal it was imperative for the glycobioinformatics community to agree on encoding formats and ensure robustness in the frameworks.

Since the exchange interface (REST) and protocol are independent of the database backend, the WGGDS guidelines can be easily incorporated and extended by other databases. Web services enable researchers to access data and provide a framework for programmers to build applications without installing and maintaining the necessary databases.

#### *Bridging glycobioinformatics and bioinformatics using RDF*

Semantic Web approaches are based on common formats that enable the integration and aggregation of data from multiple resources, which potentially offers a means to solve data compatibility issue in the glycomics space. The Semantic Web is a growing area of active research and growth in the life sciences field, which has the ability to improve bioinformatics analyses by leveraging the vast stores of data accumulated in web-accessible resources (e.g., Bio2RDF [40]). A range of commonly accessed databases such as UniProtKB has adopted the resource description framework (RDF) [41] as a format to support data integration and more sophisticated queries.

Several database projects in Japan have been involved in adopting RDF such as PDBj [42] or JCGGDB [43] as a part of the Integrated Database Project http://lifesciencedb.jp that focuses on data integration of heterogeneous datasets to provide users with a comprehensive data resource that can be accessible from a single endpoint. In order to efficiently implement RDF solutions, the existing database providers must agree on a standard for representing glycan structure and annotation information. For that purpose, the developers of major glycomics databases including BCSDB [17], GlycomeDB, JCGGDB, GLYCOSCIENCES.de and UniCarbKB designed a draft standard and prototype implementation of the RDF generation during BioHackathon 2012 http://2012.biohackathon.org.

GlycoRDF is a future-thinking collaborative effort that is addressing the requirement for sophisticated data mashups that answer complex research questions. It also allows the integration of information across different -omics, a potential that is demonstrated by the adoption of Semantic Web technologies in other fields including proteomics and genomics. The GlycoRDF innovative solution requires the harvesting of knowledge from multiple resources. Here, initial activities have focused on providing normalised RDF documents sourced from the wealth of information provided by the partners spanning structural and experimental data collections. The developers involved in this project released the first version of GlycoRDF in 2013 [48].

## Discussion

In the last few years, small collaborative projects have started between international glycobioinformatics researchers, with very limited funding, but these are slowly transforming the way glyco-related data is shared and queried. Information is getting more centralised at a technology level. Cooperation started informally at the 1^st ^Beilstein Symposium on Glyco-Bioinformatics (2009, Postdam, Germany, http://www.beilstein-institut.de/en/symposia/overview/glyco-bioinformatics, and became more structured during the 3^rd ^Warren workshop (2010, Gothenburg, Sweden, http://www.biomedicine.gu.se/biomedicine/Charles_Warren_Workshop_III. The 2^nd ^Beilstein symposium on Glyco-Bioinformatics (2011, Postdam, Germany, http://www.beilstein-institut.de/en/symposia/overview/2-glyco-bioinformatics provided an opportunity to reinforce the UniCarbKB consortium and led to the publication of a *Viewpoint *article [23] suggesting a roadmap for glycobioinformatics. At this stage, further input into WGGDS was achieved by common work on universal formats and guidelines that provide for easier integration and interoperability between glycobioinformatics applications and the data stored in the partner databases. At the 4^th ^Warren workshop (2012, Athens, Georgia, USA, http://glycomics.ccrc.uga.edu/warren-workshop), definite steps towards adopting standards were manifest and implemented in the manuscript submission process of the journal of Molecular and Cellular Proteomics http://www.mcponline.org. These events undoubtedly strengthened the coherence of glycobioinformatics initiatives. We expect that projects like MIRAGE will help drive the adoption of data standards. The next step should focus on analytical formats along the lines of the widely used MS pepXML in proteomics.

GlycomeDB and UniCarbKB are examples of initiatives that can address the issues mentioned in the first section of this paper. GlycomeDB is currently the most comprehensive and unified resource for carbohydrate structures. It integrates the structural and taxonomic data of all major public carbohydrate databases including CarbBank, KEGG, CFG, GlycoBase, BCSDB, GLYCOSCIENCES.de as well as carbohydrates contained in the Protein Data Bank (PDB). GLYCOSCIENCES.de adds information on 3D structures of glycoproteins and protein-carbohydrate complexes from the PDB as well as tools to validate and statistically analyse these data [44,45]. UniCarbKB is an informatics framework for the storage and the analysis of high-quality data collections on glycoconjugates, including informative meta-data and annotated experimental datasets. While it is still in the early development phases, this scalable web-friendly framework, at this stage, integrates curated glycan structural information and PubMed references from GlycoSuiteDB and EUROCarbDB, and experimental MS/MS data from UniCarb-DB. Information relevant to glycoproteins, notably the inclusion of glycosylated structures localised in different tissues and on different proteins, as sourced from literature mining, will bridge to the proteomics knowledgebases. Linking this information with curated data on structures recognised by bacteria and lectins as described for instance in SugarBind [46] or by glycoarray data (CFG) allows deeper mining of the functional role of glycans.

The usability of GlycomeDB and UniCarbKB sets the basis for tackling the second section of this paper as each -omics specialty comes with a bioinformatics toolbox for analysing high-throughput data. Furthermore, in the vast majority of cases, the interpretation of this data is related to gene sequences. Indeed, popular and established bioinformatics databases are sequence-centred, so that straight or translated DNA sequences constitute the fundamental piece of information around which all other useful properties or data types are organised (gene expression, protein structure, etc.). The recent move towards Systems Biology has confirmed the status of DNA/RNA/protein sequence as the element minimally shared by each -omics domain. In this context, the systematic investigation of glycan expression profiles obviously needs to be recorded with the associated glycoproteins and mapped onto amino acid sequences. This will enable further exploration of the subtle differences characterising pathological or any other specific conditions in which glycans are expressed and prevent, modulate or facilitate protein recognition and binding.

Overall, the international consortia involved in the cited projects are thereby attempting to bring together the many disconnected islands of glycobiological information in a standardised open access framework, aiming in the near future, to automatically mashup data from many resources - opening glycomics to the general scientific community.

## Conclusions

In this paper originally introduced at NETTAB'12 [47], we have first diagnosed the causes of the slow development of glycobioinformatics and the "objective" difficulties encountered in defining adequate formats for representing complex entities. We have then suggested three directions for attending to the listed issues in relation to twenty years of mixed results in developing glycobioinformatics resources.

We first advocate setting, and complying with, standards as a minimum requirement for planning the future of automated processing and analysis of glycans. We secondly embark on several programmes for bridging glycomics with other -omics following different strategies. Finally, we show by co-authoring this paper and collaborating in consortia that these initiatives should be developed and supported by a cohesive community if we wish to successfully meet the goal of integration.

The overall aim of new or improved and integrated resources is to access, query and mine existing glycobioinformation in various and complementing ways. These tools, designed to connect with other -omics information, are destined to support research in analytical glycobiology in the context of whole systems biology. They should give rise to enhanced methods for the prediction of protein function and interactions and the continued development of these resources will enable the real understanding of biological processes.

API: Application Programming Interface;

CFG: Consortium for Functional Glycomics;

IUPAC: International Union of Pure and Applied Chemistry;

KCF: KEGG Chemical Function;

MS: Mass Spectrometry;

OWL-DL: web ontology language and description logic;

RDF: Resource Description Framework;

REST: Representational State Transfer;

WGGDS: Working Group on Glycomics Database Standards;

## Competing interests

The authors declare that they have no competing interests.

## Authors' contributions

MPC developed GlycoBase v1 and develops UniCarbKB with the assistance of JZ and overseen by NHP and FL. RR developed GlycomeDB. WSY developed the GlycO ontology. TL maintains GLYCOSCIENCES.de and MonosaccharideDB. JM develops SugarBind overseen by FL and bridges to UniCarbKB. MPC and CAH develop UniCarb-DB overseen by NK and PMR in collaboration with NHP and FL. KFAK develops and oversees RINGS with the assistance of YA. DD developed GlycoWorkBench and GlycanBuilder overseen by SMH. GC develops GlycoBase v3 overseen by PMR. HN oversees JCGGDB in collaboration with KFAK. FL drafted this manuscript with the assistance of RR and MPC. All other authors read, possibly corrected and approved the submitted version.

## Declarations

The publication costs for the article are covered by the Swiss National Science Foundation grant #31003A_141215.

This article has been published as part of *BMC Bioinformatics *Volume 15 Supplement 1, 2014: Integrated Bio-Search: Selected Works from the 12th International Workshop on Network Tools and Applications in Biology (NETTAB 2012). The full contents of the supplement are available online at http://www.biomedcentral.com/bmcbioinformatics/supplements/15/S1.
